# Risk factors for *Clostridioides difficile* infection and colonization among patients admitted to an intensive care unit in Shanghai, China

**DOI:** 10.1186/s12879-019-4603-1

**Published:** 2019-11-11

**Authors:** Yingchao Cui, Danfeng Dong, Lihua Zhang, Daosheng Wang, Cen Jiang, Qi Ni, Chen Wang, Enqiang Mao, Yibing Peng

**Affiliations:** 1 Department of Laboratory Medicine, Ruijin Hospital, Shanghai Jiao Tong University School of Medicine, No. 197 Ruijin ER Road, Shanghai, 200025 China; 2 Faculty of Medical Laboratory Science, Shanghai Jiao Tong University School of Medicine, No. 197 Ruijin ER Road, Shanghai, 200025 China; 3 Department of Laboratory Medicine, Xinhua Hospital, Shanghai Jiao Tong University School of Medicine, No. 1665 Kongjiang Road, Shanghai, 200092 China; 4 Department of Emergency Intensive Care Unit, Ruijin Hospital, Shanghai Jiao Tong University School of Medicine, No. 197 Ruijin ER Road, Shanghai, 200025 China

**Keywords:** *Clostridioides difficile* infection, *Clostridioides difficile* colonization, Risk factors, Genotyping, Intensive care unit

## Abstract

**Background:**

*Clostridioides difficile* is considered the main pathogen responsible for hospital-acquired infections. This prospective study determined the prevalence, molecular epidemiological characteristics, and risk factors for *C. difficile* infection (CDI) and *C. difficile* colonization (CDC) among patients in the intensive care unit (ICU) of a large-scale tertiary hospital in China, with the aim of providing strategies for efficient CDI and CDC prevention and control.

**Methods:**

Stool samples were collected and anaerobically cultured for *C. difficile* detection. The identified isolates were examined for toxin genes and subjected to multilocus sequence typing. Patients were classified into CDI, CDC, and control groups, and their medical records were analyzed to determine the risk factors for CDI and CDC.

**Results:**

Of the 800 patients included in the study, 33 (4.12%) and 25 (3.12%) were identified to have CDI and CDC, respectively. Associations with CDI were found for fever (OR = 13.993), metabolic disorder (OR = 7.972), and treatment with fluoroquinolone (OR = 42.696) or combined antibiotics (OR = 2.856). CDC patients were characterized by prolonged hospital stay (OR = 1.137), increased number of comorbidities (OR = 36.509), respiratory diseases (OR = 0.043), and treatment with vancomycin (OR = 18.168). Notably, treatment with metronidazole was found to be a protective factor in both groups (CDI: OR = 0.042; CDC: OR = 0.013). Eighteen sequence types (STs) were identified. In the CDI group, the isolated strains were predominantly toxin A and toxin B positive (A + B+) and the epidemic clone was genotype ST2. In the CDC group, the dominant strains were A + B+ and the epidemic clone was ST81.

**Conclusions:**

The prevalences of CDC and CDI in our ICU were relatively high, suggesting the importance of routine screening for acquisition of *C. difficile*. Future prevention and treatment strategies for CDC and CDI should consider hospital stay, enteral nutrition, underlying comorbidities, and use of combined antibiotics. Moreover, metronidazole may be a protective factor for both CDI and CDC, and could be used empirically.

## Background

*Clostridioides difficile* is a Gram-positive spore-forming anaerobic bacterium listed as the leading cause of hospital-acquired diarrhea in many developed countries [1]. The pathogen secretes two main toxins, toxin A and toxin B, that mediate *C. difficile*-associated colitis and diarrhea [2]. The incidence of *C. difficile* infection (CDI) is steadily increasing worldwide and its mortality rate has risen accordingly [3, 4]. A previous report stated that the number of hospitalized patients with CDI in the United States has more than doubled during the last decade [5]. A similar situation is present in some Asian countries [6, 7], leading to prolonged hospital stays and higher costs for intensive care units (ICUs) and bringing significant economic burdens.

*C. difficile* can colonize individuals without causing detectable symptoms of infection. Such asymptomatic *C. difficile*-colonized patients may present a potential risk to other susceptible individuals by acting as infection reservoirs [8, 9]. Thus, it is considered that asymptomatic *C. difficile*-colonized patients may serve as potential vehicles for *C. difficile* transmission in medical settings [10], where there is a significantly higher risk of CDI [11]. The global spread of emerging hypervirulent toxigenic strains is of particular concern [12].

In a previous study, patients in intensive care units (ICUs) mainly received antimicrobial therapy and had comorbidities [13]. CDI patients in ICUs were reported to have prolonged hospital stays [14, 15], high hospital costs [16], and high mortality rates [17]. The current prevalence of CDI among ICU patients was estimated at 0.4–4% [18]. Furthermore, about 10–20% of ICU patients were colonized with *C. difficile* without any symptoms of infection [18]. Therefore, the presence of *C. difficile* may have a particular impact on the morbidity and mortality of patients in ICUs.

The incidence of toxigenic CDI or *C. difficile* colonization (CDC) among ICU patients in China remains largely uninvestigated. In addition, little is known about the epidemiology of strains in terms of typing, or about the in-depth risk factors. Therefore, this prospective study aimed to provide a better understanding of the prevalence, molecular epidemiological characteristics, and risk factors for CDI and CDC among patients in the ICU of a large-scale teaching hospital in China.

## Methods

### Study design, case definitions, and data collection

We conducted a prospective study on adult patients admitted to our ICU, an 18-bed department in Shanghai Ruijin Hospital, from January 2015 to June 2017. Patients were screened for *C. difficile* within 48 h of admission [19], and subsequently tested every week or at onset of diarrhea. The surveillance continued until patients died or were discharged from hospital. The study was approved by the Ethics Committee of Ruijin Hospital in Shanghai, China.

According to European guidelines [20], CDI was defined as the symptom of diarrhea and laboratory findings for toxigenic *C. difficile*, while CDC was defined as positivity for toxigenic *C. difficile* but no diarrhea [21]. To reduce the influence of confounding factors, we chose *C. difficile*-negative patients with diarrhea as controls for CDI and those without diarrhea as controls for CDC. The control groups were randomly selected from ICU patients who were admitted to the hospital during the same time period but had no history of CDI or CDC in the previous 8 weeks.

For all patients in the study, we recorded their demographic data and clinical characteristics, including duration of hospital stay, mortality, surgery (in previous 6 months), history of antibiotic use or gastric acid suppressants (for 1 month before onset of diarrhea in CDI patients and their controls, and 1 month before CDC development in CDC patients and index hospital stay in their controls), and enteral nutrition. Primary disease diagnoses were divided into six major categories: gastrointestinal disease, respiratory disease, cardiovascular disease, renal disease, neurological disease, and metabolic disorders including diabetes, hypertension, or hyperlipidemia. For laboratory test indices, body temperature, leukocyte count, serum albumin level, and serum creatinine level were measured. All laboratory indicators were recorded when patients were diagnosed with CDI or CDC. For patients in the two control groups, related laboratory indicators were tested on admission to hospital.

### *C. difficile* strain isolation and collection

Stool samples were collected from ICU patients within a set time period, plated onto *C. difficile* agar base supplemented with norfloxacin and moxalactam (Oxoid Ltd., Basingstoke, UK), and cultured anaerobically at 37 °C for 48–72 h. Colonies were identified by morphological features, latex agglutination test (*C. difficile* Agglutination Test Kit; Oxoid Ltd.), and *gluD* gene detection. Feces and *C. difficile* isolates were also subjected to toxin A/B detection by enzyme-linked fluorescence assay with a VIDAS automatic analyzer (Biomerieux, Marcy-l’Etoile, France) [22–24].

### Multilocus sequence typing (MLST)

MLST was performed for genotyping of *C. difficile* strains. For this, DNA was isolated using a DNA extraction kit (Sangon Biotech, Shanghai, China). Next, seven housekeeping genes (*adk*, *atpA*, *dxr*, *glyA*, *recA*, *sodA*, *tpi*) were amplified from all strains and sequenced as described previously [25]. The obtained sequences were aligned with sequences in the MLST database (*http://pubmlst.org/clostridium* *difficile*).

### Data analysis

Continuous variables were expressed as median and standard deviation, and compared by Student’s *t*-test. Categorical variables were presented as frequency or percentage, and compared by the chi-square test or Fisher’s exact test. Univariate analyses were performed to evaluate the potential risk factors relevant to the cases. The statistically significant variables from the univariate analyses were then included in a multivariate logistic regression model. The results of the logistic regression analysis were presented as odd ratio (OR) and 95% confidence interval (95% CI).

All analyses were performed with Statistical Program for Social Sciences (SPSS) version 22.0 for Windows software. Values of *P* < 0.05 were considered statistically significant.

## Results

### Patient population

**Fig. 1:**
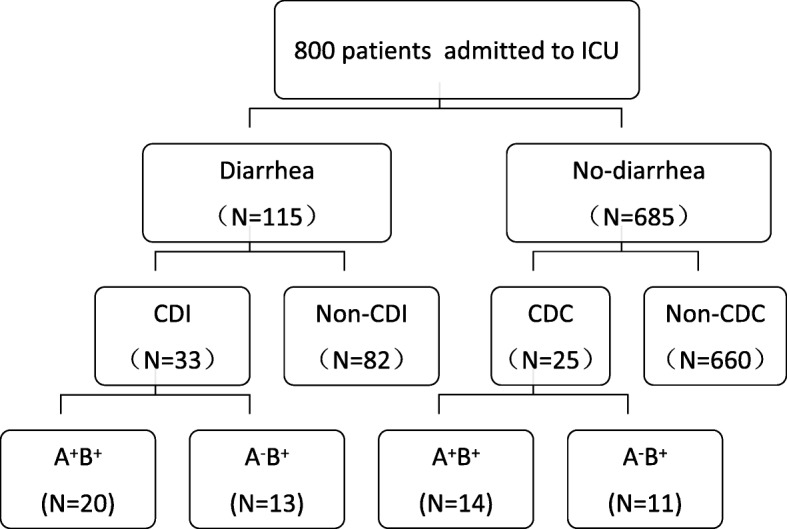
Study flowchart of CDI and CDC patients among the ICU patients. A total of 800 patients were included in the study and divided into two groups according to whether or not they had diarrhea. Further grouping was performed according to the detection of *C. difficile* and toxin genes

As shown in Figs. 1, 800 adult patients were admitted to the ICU during the study period. Of these, 115 developed diarrhea and 33 (28.70%) were identified to have CDI. Another 25 toxigenic *C. difficile* strains were isolated from patients without diarrhea, and defined as CDC cases. The overall prevalence of CDI and CDC was 4.12 and 3.12%, respectively, and all cases were healthcare facility-associated. One patient had recurrence of infection, one patient transitioned from CDC to CDI, and two patients had infections with two different types. The CDI and CDC patients had median age of 54.15 and 62 years, male proportion of 66.7 and 68%, and period from admission until positive test result of 17.06 ± 12.97 and 31.16 ± 33.85 days, respectively. Neither age nor sex showed any significant difference between the groups. To assess the potential risk factors and clinical characteristics, 66 non-CDI and 50 non-CDC patients were included as control groups.

### Clinical characteristics and risk factors for ICU patients with CDI

As illustrated in Table 1, univariate analyses were conducted to determine the differences between the CDI group and the non-CDI control group in terms of clinical characteristics, diagnosis, and treatment. The CDI patients were more likely to suffer from fever (OR, 6.786; 95% CI, 2.634–17.483; *P* < 0.001) and metabolic disorders (OR, 3.28; 95% CI, 1.363–7.893; *P* < 0.05) than the non-CDI patients. The CDI patients also had a larger number of comorbidities (P < 0.05). Compared with the non-CDI patients, the CDI patients more frequently received enteral feeding (78.8% vs. 50%; OR, 3.714; 95% CI, 1.416–9.74), antiviral drugs (15.2% vs. 1.52%; OR, 11.607; 95% CI, 1.296–103.948), and fluoroquinolone (21.2% vs. 3%; OR, 8.615; 95% CI, 1.678–44.247) during their hospitalization (*P* < 0.05). Furthermore, a larger proportion of CDI patients were administered more than one type of antibiotic (P < 0.05). To further assess the potential risk factors for CDI, a multivariable logistic regression analysis was performed. The results showed that fever, metabolic disorder, and treatment with fluoroquinolone or combined antibiotics were risk factors associated with development of CDI among ICU patients. However, treatment with metronidazole was found to be a protective factor (OR, 0.042; 95% CI, 0.006–0.288; *P* = 0.001).

**Table 1:** Univariate and multivariate analyses of the demographic, clinical characteristics, and risk factors in CDI groups

Characteristics	CDI group (*n* = 33)	non-CDI group (*n* = 66)	Univariate Analysis		Multivariable logistic regression Analysis	
	n(%)/mean ± SD	n(%)/mean ± SD	OR (95% CI)	*P* value	OR (95% CI)	*P* value
Male	22 (66.7)	39 (60)	0.722 (0.301–1.732)	0.465		
Age (mean ± SD)	54.15 ± 20.89	58.97 ± 14.87	–	0.242		
Clinical features						
Hospital duration (days) (mean ± SD)	35.39 ± 27.61	30.08 ± 33.11	–	0.429		
Fever (≥ 38 °C)	19 (57.6)	11 (16.7)	6.786 (2.634–17.483)	< 0.001*	13.993 (3.292–59.472)	< 0.001*
Leukocyte count (10^9^ /L) (mean ± SD)	9.79 ± 5.35	10.61 ± 6.24	–	0.992		
Serum albumin (g/L) (mean ± SD)	30.30 ± 6.02	29.97 ± 7.01	–	0.816		
Serum creatinine rise> 50% (μmol/L)	2 (6.06)	12 (18.18)	–	0.103		
Mortality	6 (18.2)	11 (16.7)	1.111 (0.371–3.325)	0.851		
Classification of primary diagnosis						
Gastrointestinal	29 (87.9)	55 (83.8)	1.45 (0.424–4.959)	0.552		
Respiratory	12 (36.4)	26 (39.4)	0.879 (0.37–2.086)	0.770		
Cardiovascular	6 (18.2)	14 (21.2)	0.825 (0.285–2.39)	0.723		
Renal	5 (15.2)	17 (25.8)	0.515 (0.171–1.546)	0.231		
Neurologic	8 (24.2)	9 (13.6)	2.027 (0.701–5.862)	0.187		
Metabolic disorders	22 (66.7)	25 (37.9)	3.28 (1.363–7.893)	0.007*	7.972 (1.767–35.971)	0.007*
NO. of comorbidities^a^						
1–2	15 (45.5)	43 (65.2)	–	0.037*		
3–4	16 (48.5)	21 (31.8)				
≥5	2 (6.1)	1 (1.5)				
Treatments and procedures						
Surgical intervention	7 (21.2)	15 (22.7)	0.915 (0.332–2.523)	0.864		
Enteral feeding	26 (78.8)	33 (50)	3.714 (1.416–9.74)	0.006*		
PPI use	17 (51.5)	43 (65.2)	0.568 (0.243–1.330)	0.191		
Antibiotics use	31 (93.9)	57 (86.4)	2.447 (0.497–12.042)	0.258		
Antiviral drugs	5 (15.2)	1 (1.52)	11.607 (1.296–103.948)	0.007*		
Antifungal agents	6 (18.2)	6 (9.1)	2.222 (0.657–7.522)	0.191		
Cephalosporin	9 (27.3)	26 (29.4)	0.577 (0.232–1.435)	0.234		
Fluoroquinolone	7 (21.2)	2 (3.0)	8.615 (1.678–44.247)	0.003*	42.696 (3.895–468.058)	0.002*
Carbapenem	24 (72.7)	35 (53.0)	2.362 (0.955–5.843)	0.060		
Vancomycin	10 (20.3)	13 (19.7)	1.773 (0.68–4.624)	0.239		
Metronidazole	5 (15.2)	22 (33.3)	0.357 (0.121–1.052)	0.056	0.042 (0.006–0.288)	0.001*
NO. of antibiotics received^a^						
0	2 (6.1)	9 (13.6)	–	0.024*	2.856 (1.362–5.99)	0.005*
1–2	20 (6.1)	48 (72.7)				
≥3	11 (33.3)	9 (13.6)				

Numerical data are shown as mean ± SD, and categorical data are described as frequency (percentage)

*P < 0.05

^a^The indicated variables were made categorical, and analyzed for differences between the two groups by the Cochran–Armitage trend test

### Clinical characteristics and risk factors for ICU patients with CDC

The median hospital stay for CDC patients was 62 days and significantly longer than that for non-CDC patients (*P* < 0.05). This difference was further verified by the multivariable logistic regression model. Colonization of *C. difficile* did not cause any significant differences in laboratory test indices, including leukocyte count and serum albumin or creatinine levels. However, patients with respiratory or neurological disease were more likely to acquire asymptomatic CDC. Number of comorbidities was a potential risk factor for CDC patients (OR, 36.509; 95% CI, 2.602–512.183; *P* = 0.08). For treatment procedures, surgical intervention, enteral feeding, antifungal agent usage, and carbapenem medication were more frequently found in CDC patients than in non-CDC patients (*P* < 0.05). The multivariable model analysis showed that vancomycin was an independent risk factor (OR, 18.168; 95% CI, 1.036–318.503; *P* = 0.047), while metronidazole was a protective factor (OR, 0.013; 95% CI, 0–0.512; *P* = 0.021) for CDC (Table 2).

**Table 2:** Univariate and multivariate analyses of the demographic, clinical characteristics, and risk factors in CDC groups

Characteristics	CDC group (*n* = 33)	non-CDC group (*n* = 66)	Univariate Analysis		Multivariable logistic regression Analysis	
	n(%)/mean ± SD	n(%)/mean ± SD	OR (95% CI)	*P* value	OR (95% CI)	*P* value
Male	17 (68.0)	31 (62.0)	0.768 (0.278–2.121)	0.610		
Age (mean ± SD)	62 ± 18.93	59.06 ± 10.54	–	0.660		
Clinical features						
Hospital duration (days) (mean ± SD)	61.28 ± 66.12	16.98 ± 11.48	–	0.003*	1.137 (1.05–1.23)	0.002*
Fever (≥ 38 °C)	9 (36.0)	7 (14.0)				
Leukocyte count (10^9^ /L) (mean ± SD)	9.84 ± 5.32	8.75 ± 5.07	–	0.389		
Serum albumin (g/L) (mean ± SD)	31.12 ± 5.55	33.80 ± 10.53	–	0.238		
Serum creatinine rise> 50%(μmol/L)	2 (8.0)	5 (10)	–	0.779		
Mortality	3 (12.0)	2 (4.0)	3.273 (0.51–21.002)	0.190		
Classification of primary diagnosis						
Gastrointestinal	18 (72.0)	30 (60.0)	1.714 (0.606–4.852)	0.307		
Respiratory	15 (60.0)	19 (38.0)	2.447 (0.916–6.541)	0.071	0.043 (0.002–0.969)	0.048*
Cardiovascular	7 (28.0)	18 (36.0)	0.691 (0.243–1.969)	0.488		
Renal	9 (36.0)	15 (30.0)	1.313 (0.475–3.626)	0.600		
Neurologic	6 (24.0)	3 (6.0)	4.947 (1.121–21.838)	0.024*		
Metabolic disorders	12 (48.0)	26 (52.0)	0.852 (0.326–2.227)	0.744		
NO. of comorbidities						
1–2	13 (52.0)	33 (66.0)	–	0.139	36.509 (2.602–512.183)	0.008*
3–4	9 (36.0)	17 (34.0)				
≥5	3 (12.0)	0 (0)				
Treatments and procedures						
Surgical intervention	10 (40.0)	4 (8.0)	7.667 (2.094–28.068)	0.001*		
Enteral feeding	16 (64.0)	16 (32.0)	3.778 (1.376–10.372)	0.008*		
PPI use	8 (32.0)	25 (50.0)	0.471 (0.172–1.288)	0.139		
Antibiotics use	24 (96.0)	41 (82.0)	5.268 (0.628–44.178)	0.093		
Antiviral drugs	2 (8.0)	3 (6.0)	1.362 (0.213–8.729)	0.743		
Antifungal agents	7 (28.0)	4 (8.0)	4.472 (1.166–17.146)	0.021*		
Cephalosporin	9 (36.0)	24 (48.0)	0.609 (0.227–1.636)	0.324		
Fluoroquinolone	5 (20.0)	8 (16.0	1.313 (0.381–4.525)	0.666		
Carbapenem	18 (70.0)	21 (42.0)	3.551 (1.258–10.027)	0.014*		
Vancomycin	12 (48.0)	7 (14.0)	5.67 (1.851–17.374)	0.001*	18.168 (1.036–318.503)	0.047*
Metronidazole	2 (8.0)	12 (24.0)	0.275 (0.056–1.342)	0.094	0.013 (0–0.512)	0.021*
NO. of antibiotics received						
0	1 (4.0)	9 (18.0)	–	0.076		
1–2	16 (64.0)	33 (66.0)				
≥3	8 (32.0)	8 (16.0)				

Numerical data are shown as mean ± SD, and categorical data are described as frequency (percentage)

*P < 0.05

^a^The indicated variables were made categorical, and analyzed for differences between the two groups by the Cochran–Armitage trend test

### Molecular characteristics of *C. difficile*

The toxin types were detected for the 58 *C. difficile* strains isolated from the CDI and CDC patients. In total, 34 (58.6%) were A + B+ (positive for both *tcdA* and *tcdB*) and 24 (41.3%) were A − B+ (negative for *tcdA* and positive for *tcdB*). Specifically, 20 (60.6%) were A + B+ and 13 (39.4%) were A − B+ in the CDI group, while 14 (56%) were A + B+ and 11 (44%) were A − B+ in the CDC group.

**Fig. 2:**
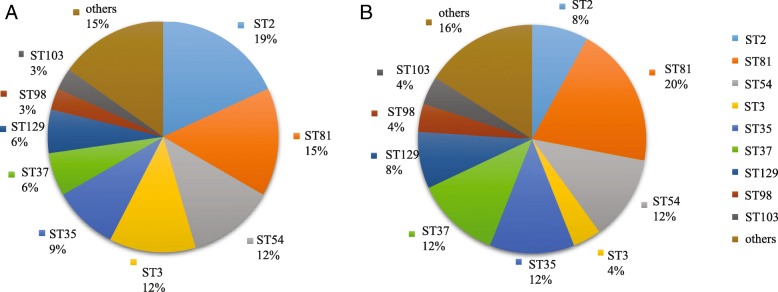
Proportions of STs of *C. difficile* strains isolated from patients in the ICU. **a** Proportions of STs in the CDI group. **b** Proportions of STs in the CDC group

MLST was also performed on the strains, and 18 sequence types (STs) were identified (Fig. 2). In the CDI group, ST2, ST81, ST54, and ST3 were the major STs, constituting 19, 15, 12, and 12% of the strains, respectively. In the CDC group, ST81, ST35, ST37, and ST54 were the dominant types, accounting for 20, 12, 12, and 12% of the strains, respectively.

**Fig. 3:**
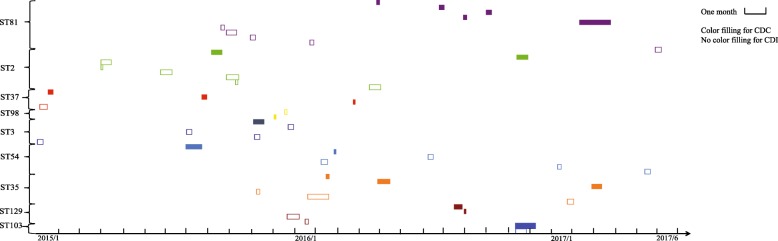
Time-space cluster map of different STs from CDI and CDC patients in the ICU. Each small box represents the duration from date of detection of *C. difficile* in the stool of a hospitalized ICU patient to the date at which *C. difficile* was no longer detectable

Based on the STs of the strains, a map was constructed to compare the temporospatial relationships of the same STs in the two groups during the study period (Fig. 3). Two overlaps were detected within the CDI group for ST2 and one overlap was detected between the CDI and CDC groups for ST103. No overlaps were detected among the other STs.

## Discussion

During recent decades, there has been a continuous increase in CDI and CDC cases among hospitalized patients in many medical settings [1, 6, 7, 21]. Patients in ICUs often suffer from various comorbidities, which greatly increase the risk of developing CDI and lead to difficulties in treatment of the underlying medical conditions [26]. A review described that approximately 2% of ICU patients suffered from CDI, which was significantly higher than the 0.9% of patients on general wards [27]. In our study, we found that the prevalence of CDI was 4.12%, and much higher than that in most studies reported from European countries [27]. Furthermore, 28.7% of ICU patients with diarrhea developed CDI, which was much higher than the 8% reported in another Chinese study [28]. Meanwhile, the detection rate of CDC in our study was 3.12%, and relatively lower than the 7% reported in a retrospective study from Kuwait [29]. Above all, the prevalences of CDI and CDC varied geographically. The high acquisition of toxigenic *C. difficile* may result from increased screening and the highly sensitive detection methodology used, as well as enhanced awareness for prevention of *C. difficile*-related diseases. Moreover, it is possible that the incidence of *C. difficile* changed distinctly, especially in ICU patients.

The main reported risk factors for CDI included antibiotic exposure, age > 60 years, longer hospital stay, severe dyspepsia, history of gastric acid inhibitor use [30], enteral feeding, and proton pump inhibitor (PPI) medication [31]. ICU admission was also a common pathogenic factor [32]. In the present study, we found that medication with multiple antibiotics significantly increased the risk of developing CDI. Specifically, increased use of fluoroquinolones contributed to the incidence of CDI, as previously suggested [32, 33]. Routine interventions especially relevant for patients in ICUs, such as surgery, enteral feeding, and PPI medication, doubled the risk of CDI infection [16, 30, 34–36]. PPIs caused a change in the gastrointestinal flora, thereby creating a niche for CDC [37]. In the present study, neither history of surgery nor PPI medication were found to differ among CDI patients. Meanwhile, CDI patients were more likely to receive enteral feeding in our univariable analyses, but not in the multivariable logistic regression analysis. Regarding underlying conditions, the study revealed a significant association between occurrence of CDI and metabolic diseases. However, the mechanisms involved remain unclear and require further investigations.

For patients with CDC in our study, large differences in number of comorbidities and duration of hospital stay were detected, consistent with a previous study [38]. However, CDC rarely occurred in patients with respiratory diseases, and the underlying reason for this finding remains to be clarified. Exposure to a variety of antibiotics was a risk factor for CDI, but not for CDC. The significant discrepancy between these findings may indicate that destruction of the intestinal microbiota by antibiotic exposure is not a key feature of CDC.

For decades, metronidazole and vancomycin have been the main antimicrobial agents for treatment of CDI [39]. In treatment analyses of three randomized controlled trials comparing metronidazole and vancomycin [40–42], no significant differences were found [43, 44]. In the present study, metronidazole was identified as a protective factor against CDI, consistent with previous studies [30, 45]. However, oral vancomycin was a risk factor for CDC, in accordance with the findings of Johnson et al. [46]. These results suggest that preventive use of metronidazole may contribute to prevention of CDI and CDC, while caution is required for medication with vancomycin in the clinic.

ST2 and ST81 were the most common strain types in the CDI group and CDC group, respectively. These findings differed from the identification of ST54 as the most common genotype in a previous study [47]. In addition, neither ST1 nor ST11, which were epidemic in Western countries [48], was detected in the present study. Our map showing the temporospatial relationships among the strains revealed that *C. difficile* dispersed among normal colonized patients could be a potential source of infection, but there was still no definitive evidence demonstrating its transmission from colonized patients to other patients.

There are several limitations to our study. First, the samples were collected from a single center and may not be representative of all healthcare institutions. However, to our knowledge, this research is one of the limited studies to report the clinical and molecular characteristics of *C. difficile* among ICU patients in China. To overcome this limitation, long-term multicenter studies should be carried out in the future. Second, because ICU wards are always isolated and strictly disinfected rooms, environmental samples were not obtained and we could not fully assess *C. difficile* transmission. To identify the risk factors for development of CDI and CDC, most previous studies compared *C. difficile*-positive cases with *C. difficile*-negative cases [49–51]. However, because most negative cases had no diarrhea, the risk factors identified for CDI in these cases were unlikely to be specific. To overcome this shortcoming, we used two sets of patients with and without diarrhea as negative controls.

## Conclusions

Our study provided prospective independent comparisons of patients in an ICU and characterized the molecular epidemiology. The overall prevalence of CDI and CDC was 4.12 and 3.12%, respectively. Fever, metabolic disorders, use of fluoroquinolone, and exposure to multiple antibiotics were significantly associated with CDI. Longer hospital stay, number of comorbidities, and use of vancomycin were associated with acquisition of CDC. Regarding metronidazole, protective effects were detected for both groups. The most common epidemic strain was ST2 and ST81 in the CDI group and CDC group, respectively. The present results highlight the importance of antimicrobial stewardship and pathogen isolation for the prevention and treatment of *C. difficile*-related diseases. The role of asymptomatic carriers in the transmission of *C. difficile* requires further investigation. In conclusion, it is essential for medical staff to emphasize the importance of *C. difficile*-related diseases, especially for ICU patients.

## Data Availability

The datasets used and/or analyzed during the current study are available from the corresponding author on reasonable request.

## Abbreviations

CDC: *Clostridioides difficile* colonization
CDI: *Clostridioides difficile* infection
ICU: Intensive care unit
MLST: Multilocus sequence typing
ST: Sequence type
